# Class A Scavenger Receptors Are Used by Frog Virus 3 During Its Cellular Entry

**DOI:** 10.3390/v11020093

**Published:** 2019-01-23

**Authors:** Nguyen T. K. Vo, Matthew Guerreiro, Amulya Yaparla, Leon Grayfer, Stephanie J. DeWitte-Orr

**Affiliations:** 1 Department of Health Sciences, Wilfrid Laurier University, Waterloo, ON N2L 3C5, Canada; nathanntkvo@gmail.com; 2 Department of Biology, Wilfrid Laurier University, Waterloo, ON N2L 3C5, Canada; guer5930@mylaurier.ca; 3 Department of Biological Sciences, George Washington University, Washington, DC 20052, USA; ayap@gwmail.gwu.edu (A.Y.); leon_grayfer@email.gwu.edu (L.G.)

**Keywords:** frog virus 3, ranavirus, iridovirus, class A scavenger receptors, viral entry, tadpole, macrophage, *Xenopus*, *Lithobates*, SR-AI

## Abstract

Frog virus 3 (FV3) is the type species of the genus *Ranavirus* (family Iridoviridae). FV3 and FV3-like viruses are globally distributed infectious agents with the capacity to replicate in three vertebrate classes (teleosts, amphibians, and reptiles). At the cellular level, FV3 and FV3-like viruses can infect cells from virtually all vertebrate classes. To date, the cellular receptors that are involved in the FV3 entry process are unknown. Class A scavenger receptors (SR-As) are a family of evolutionarily conserved cell-surface receptors that bind a wide range of chemically distinct polyanionic ligands and can function as cellular receptors for other DNA viruses, including vaccinia virus and herpes simplex virus. The present study aimed to determine whether SR-As are involved in FV3 cellular entry. By using well-defined SR-A competitive and non-competitive ligand-blocking assays and absolute qPCR, we demonstrated that the SR-A competitive ligands drastically reduced the quantities of cell-associated viral loads in frog cells. Moreover, inducing the expression of a human SR-AI in an SR-A null cell line significantly increased FV3–cell association. Together, our results indicate that SR-As are utilized by FV3 during the cellular entry process.

## 1. Introduction

Ranaviruses (RVs) are members of the family *Iridoviridae* with large double-stranded DNA (dsDNA) genomes and are currently considered to be important emerging pathogens of cold-blooded vertebrates around the globe [1]. As of 2015, RVs have been identified on six continents and ranavirus infections have been documented in 105 amphibian species (18 families), 41 fish species (22 families), and 29 reptile species (12 families) [2]. Notably, RVs are associated with mass die-offs of animals, both in the wild and in captivity [2]. Because many amphibian species possess poorly understood life cycles, reside in poorly accessible habitats, and may harbor asymptomatic/sub-lethal RV infections, the number of RV-susceptible animals and the prevalence of the disease are likely underestimated.

Frog virus 3 (FV3) is the type species of the genus *Ranavirus*. Geographically, FV3 and FV3-like viruses are widely distributed and have the capacity to infect three taxonomically distinct vertebrate classes [2]. The first known isolate of FV3 was extracted from a diseased Northern leopard frog (*Lithobates pipiens*) in the USA by Granoff et al. (1965) [3]. Since then, FV3 and FV3-like viruses have been found in other frogs, salamanders, fish, and reptiles [2]. Some FV3-permissive frogs from the North American landscape include wood frogs (*Lithobates sylvaticus*), green frogs (*Lithobates clamitans*), bullfrogs (*Lithobates catesbeianus*), and American toads (*Anaxyrus americanus*) [2,4]. Based on work using the relatively less FV3-suceptible *Xenopus laevis* frog model, it has been shown that frog macrophages (Mϕs) are crucial to both the FV3 infection strategy as well as the immune defenses against this pathogen [5,6]. Interestingly, *X. laevis* Mϕs differentiated with either colony-stimulating factor 1 (CSF-1) or interleukin-34 (IL-34) Mϕ growth factors are highly susceptible and resistant to FV3, respectively [7]. 

FV3, and presumably at least some of the other iridoviruses, are infectious as both enveloped and naked virions [8,9,10]. Enveloped FV3 virions are more infectious than their naked counterparts [11]. Enveloped FV3 appear to enter cells via receptor-mediated endocytosis that is associated with clathrin-coated pits [12,13]. Conversely, naked FV3 virions primarily penetrate cells by fusion at the plasma membrane followed by injection of their nucleocapsid content into the cytoplasm, but they may sporadically enter cells via a receptor-mediated endocytosis-like mechanism [12,13,14,15]. Soft-shelled turtle iridovirus (STIV, a reptilian ranavirus that is very closely related to FV3) and Singapore grouper iridovirus (SGIV, a fish ranavirus that is genetically divergent from FV3 and STIV [16]) enter cells via clathrin-mediated endocytosis (like FV3) as well as macropinocytosis (unlike FV3) [17,18]. By contrast, Guo et al. recently showed that tiger frog virus (an amphibian ranavirus that is genetically similar to FV3 and STIV) and infectious spleen and kidney necrosis virus (a fish iridovirus belonging to the genus *Megalocytivirus*) enter cells via mechanisms that share many features with caveolae-mediated endocytosis [19,20]. The identity and the number of the cell surface receptors that dictate RV tropisms and facilitate viral entry are currently unknown.

The very broad host range and cellular tropisms of RVs may well reflect the nature of their cellular receptors. Indeed, RVs can be internalized into fish, amphibian, reptile, avian, and mammalian cells [11,13,17,19,21,22,23,24]. However, FV3 does not appear to infect invertebrate cells such as the insect Sf-9 II cell line (L. Grayfer, personal observations), suggesting that the RV cellular receptors are encoded by numerous vertebrates but are absent in invertebrates. Moreover, FV3 is capable of infecting many distinct cell types including epithelial cells, fibroblasts, myocardiocytes, endothelial cells, Mϕs, and embryonic cells [11,13,21,23,24,25], suggesting that most cell types ubiquitously express some of the FV3 cellular receptors. 

Class A scavenger receptors (SR-As) are a family of cell-surface receptors that bind a wide range of chemically distinct polyanionic ligands including acetylated low-density lipoproteins (AcLDLs), bacterial pathogen-associated molecular patterns (PAMPs), and nucleic acids [26,27,28]. There are five known members in the SR-A family: SR-AI/II, macrophage receptor with collagenous domain (MARCO), scavenger receptor A (SCARA)3, SCARA4, and SCARA5 [29,30,31,32]. SR-As are present in all vertebrates and absent in invertebrates [33], ubiquitously expressed by many distinct cell types [27,29,32,34,35], function at a wide range of temperatures, exhibit evolutionarily conserved ligand binding properties [27,34,35,36,37], and facilitate clathrin/caveola-mediated endocytosis and macropinocytosis [38,39,40]. Moreover, several SR-A members facilitate cellular entry of DNA viruses. For example, vaccinia virus (VACV) and herpes simplex virus 1 (HSV-1) bind to MARCO on the surface of human epithelial cells [41,42], while adenovirus 5 (AdV-5) binds to both MARCO and SR-AII [43,44]. Notably, competitive SR-A ligands were instrumental in defining SR-As as cellular receptors for VACV, HSV-1, and AdV-5 [41,43,45]. Together, SR-As represent likely cellular receptor candidates for RV entry.

In light of the above, in the present study we examined the putative roles of SR-As as receptors for FV3 cellular entry into an array of amphibian cell types.

## 2. Materials and Methods

### 2.1. Cell Culture Media

All growth media used for the maintenance of cell lines were supplemented with 10% fetal bovine serum (FBS, Seradigm, Mississauga, Ontario), 100 U/mL penicillin, and 100 μg/mL streptomycin (Thermo Fisher Scientific, Ottawa, ON, Canada). For *X. laevis* primary bone marrow cell cultures, amphibian phosphate buffered saline (APBS: 100 mM sodium chloride, 8 mM sodium phosphate, 1.5 mM potassium phosphate; pH 7.7), and amphibian serum-free medium (ASF: 70% Iscove’s Modified Dulbecco’s medium, 30% water) supplemented with 10% FBS, 0.25% *X. laevis* serum, 10 μg/mL gentamycin (Thermo Fisher Scientific, Waltham, MA), 100 U/mL penicillin, 100 μg/mL streptomycin (Gibco, Waltham, MA), 2.1 mg/mL NaHCO_3_, 0.21% (*v*/*v*) Primatone, 0.07% (*v*/*v*) 2-mercatoethanol, 75 μM Minimum Essential Medium (MEM) non-essential amino acids, and 35 μg/mL insulin were used as previously described [46]. All virus infection media were supplemented with 2–2.5% FBS, 100 U/mL penicillin, and 100 μg/mL streptomycin.

### 2.2. Cell Lines and Primary Macrophage Cultures

Four tadpole cell lines (BufoTad, BullTad-leg, WoodTad-rpe, and GreenTad-HF2) were used (Table 1). BufoTad and BullTad-leg were obtained from Eric Leis at the La Crosse Fish Health Center in Wisconsin. WoodTad-rpe and GreenTad-HF2 were developed at Wilfrid Laurier University in Ontario. All tadpole cell lines were grown in the modified amphibian L-15 (AL-15) growth medium (70% Leibovitz’s L-15 (Hyclone, Thermo Fisher Scientific, Ottawa, ON, Canada) and 30% water).

The fathead minnow EPC cell line was maintained in the L-15 growth medium. EPC was obtained from Dr. Niels Bols (University of Waterloo, Waterloo, ON, Canada). 

The baby hamster kidney (BHK-21) cell line was maintained in the Dulbecco’s Modified Eagle medium (DMEM; VWR, Radnor, PA, USA) with heat-inactivated FBS. The cell line was obtained from Dr. Jacques Robert (University of Rochester, Rochester, NY, USA).

The hSR-AI-A549 cell line has three vector constructs: pB-TET-SR-AI (response plasmid containing the *hSR-AI* gene), pB-CAG-rtTA (plasmid containing regulatory protein, rtTA), and pCyL43 (plasmid that encodes transposase) [28]. The cell line was maintained in the Roswell Park Memorial Institute (RMPI) 1640 growth medium with heat-inactivated FBS and 25 mM 4-(2-hydroxyethyl)-1-piperazineethanesulfonic acid (HEPES). hSR-AI expression is tightly regulated and robustly inducible by 10–1000 ng/mL doxycycline (DOX) [28]. The cell line was obtained from Dr. Karen Mossman (McMaster University, Hamilton, ON, Canada). 

Outbred adult *X. laevis* were purchased from the Xenopus 1 facility and housed and handled under strict laboratory and Institutional Animal Care and Use Committee (IACUC) regulations (Approval number 15-024). Bone marrow (BM)-derived primary macrophage (Mϕ) cultures were established as previously described [47]. In brief, following frog euthanization by tricaine methane sulfonate overdose and cervical dislocation, the femur bones were aseptically removed. The BM cells were flushed out of the femur bones with ice-cold APBS, collected, and washed with ice-cold APBS. Freshly isolated BM cells (10^5^ cells) were enumerated and cultured with recombinant CSF-1 or IL-34 (250 ng/mL; produced as previously described [7]) for 5 days prior to the SR-A ligands/FV3 experiments.

### 2.3. Routine Cell Line Maintenance 

On a weekly basis, subcultures were performed with either trypsin/EDTA or TrypLE Express (Gibco, Thermo Fisher Scientific, Ottawa, ON, Canada). Tadpole and fish cell lines were grown at 25 °C and 30 °C, respectively, at ambient atmospheric air. Mammalian cell lines were grown at 37 °C and in 5% CO_2_. 

### 2.4. FV3 Stocks

The Granoff strain of FV3 (ATCC® VR-567; [3]) was used in this study. FV3 was obtained from either Dr. Niels Bols or Dr. Jacques Robert. Either the EPC cell line or the BHK-21 cell line was used to produce the FV3 stocks [23,48]. BHK-21-derived FV3 was used for the *X. laevis* macrophage experiments, while EPC-derived FV3 was used for infections with the other frog and human cell lines. Viral titers were determined by plaque assay using 0.6% (*w*/*v*) and 1% (*w*/*v*) methylcellulose overlay on the EPC and BHK-21 cell reporters, respectively, as previously described [47].

### 2.5. SR-A Ligand Binding Blocking Assay

Three sets of SR-A competitive ligands and corresponding non-competitive ligands were used: DxSO_4_ and ChSO_4_, fucoidan and fetuin, and poly inosinic acid (pI) and poly cytidylic acid (pC). All were purchased from Sigma-Aldrich (St. Louis, MO, USA).

For experiments with tadpole cells, 5 × 10^5^–6 × 10^5^ cells were seeded per well in six-well plates and incubated with the two following sets of SR-A ligands: fucoidan/fetuin and pI/pC. All were incubated at the final concentration of 250 μg/mL in serum-free media for 30 min. Following the SR-A ligand binding blocking, the cells were inoculated with FV3 at an multiplicity of infection (MOI) of 1.0. 

For experiments with *X. laevis* BM r*Xl*CSF-1- and r*Xl*IL-34-derived Mϕs, 10,000 cells were seeded per well in 96-well plates and incubated with the two following sets of SR-A ligands: DxSO_4_/ChSO_4_ and fucoidan/fetuin. All were incubated at the final concentration of 200 μg/mL in serum-free media for 1 h. Following the SR-A ligand binding blocking, the cells were inoculated with FV3 at an MOI of 0.5. 

In all experiments, following 2 h of FV3 inoculation, cells were then harvested for DNA extraction for quantitative PCR (qPCR), to assess for the FV3 copy number as described below. For tadpole cell lines, three independent experiments were performed. For *X. laevis* primary Mϕs, cells from five individual frogs were used. Cell culture without the ligand controls were always included.

### 2.6. Induced Cell-Surface Expression of hSR-AI of hSR-AI-A549 Cells and FV3 Interaction

Cells were grown with 200 ng/mL DOX for 48 h in 75-cm^2^ flasks. Control flasks were cultured in parallel but did not receive DOX. In six-well plates, 6.6 × 10^5^ control or DOX-treated cells were seeded per well and incubated without or with DOX overnight. Cultures were then inoculated with FV3 at an MOI of 0.5 for 1 h, followed by cell harvesting for DNA extraction for qPCR for viral loads. Three independent experiments were performed. 

### 2.7. Assessment of FV3 Copy Number by Absolute qPCR

FV3 copy numbers were determined by absolute qPCR as initially described by Grayfer et al. (2014) [49]. FV3 DNA polymerase (Pol) was used as the proxy for the FV3 DNA copy number. Briefly, an FV3 vDNA Pol (ORF 60R) PCR fragment was cloned into a pGEM-T vector (Promega, Madison, WI, USA) and amplified in competent *E. coli* cells. The FV3 vDNA Pol-containing plasmids were isolated and quantified. The serially diluted concentrations of the plasmid stock were used to generate the standard curves in all absolute qPCR experiments. The forward primer was 5’-CAAGAACGTGTGCTACTCCA and the reverse primer was 5’-AGCCTCTCGTACTCTACCTTC. All absolute qPCR analyses were performed using 50 ng of total isolated DNA. The annealing temperature was 60 °C and the number cycle was 40. All qPCR experiments were performed using a CFX Connect Real-Time PCR Detection System (Bio-Rad, Mississauga, ON, Canada) and either iTaq Universal SYBR Green Supermix or SsoFast EvaGreen Supermix (Bio-Rad). 

### 2.8. Statistical Analysis

Wherever appropriate, a one-way analysis of variance (ANOVA) and Tukey’s multiple comparisons test or a Student’s t-test with Welch’s correction was performed and a 95% confidence interval was obtained using the GraphPad Prism 7.0 software (GraphPad, San Diego, CA, USA). *p*-values less than 0.05 were deemed statistically significant.

## 3. Results and Discussion

Frog cells were pre-treated with SR-A ligands followed by 2 h FV3 exposure. Tadpole cells and adult frog Mϕs were chosen as virus targets due their relative susceptibility to FV3 infection [23,50]. The tadpole cell lines tested were WoodTad-rpe from *L. sylvaticus*, GreenTad-HF2 from *L. clamitans*, BullTad-leg from *L. catesbeianus*, and BufoTad from *A. americanus*. According to our expression analyses, none of the tadpole cell lines expressed MARCO, while all of the tadpole cell lines, with the exception of the WoodTad-rpe, expressed SR-AI (Table 2). BullTad-leg, GreenTad-HF2, and WoodTad-rpe also expressed SCARA3, 4, and 5 (Table 2). Conversely, the *X. laevis* CSF-1- and IL-34-derived Mϕs expressed MARCO, SCARA3, and SCARA4, but did not express SR-AI or SCARA5 (Table 2). All SR-A gene expression was determined by RT-PCR using validated primers [35,51] based on the species-specific partial/complete SR-A sequences in the National Center for Biotechnology Information (NCBI) database. 

Positive denotes a positive SR-A transcript has been detected and sequenced. Negative denotes an SR-A transcript could not be detected by RT-PCR but the primers were successfully used to amplify a transcript in another cell line. Unknown indicates that a sequence could not be amplified in any cell line from that species.

**Figure 1:**
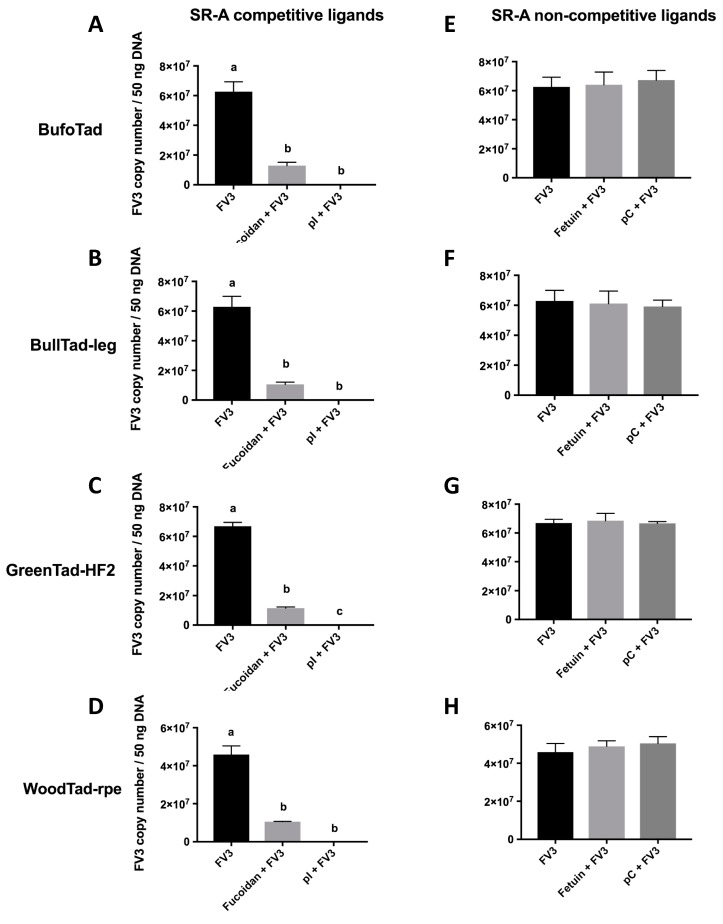
Effects of SR-A competitive and non-competitive ligands on the cell-associated FV3 loads in four tadpole cell lines. The American toad BufoTad cell line, bullfrog BullTad-leg cell line, green frog GreenTad-HF2 cell line, and wood frog WoodTad-rpe cell line were pre-treated with 250 μg/mL of the SR-A competitive ligands (fucoidan, poly inosine (pI)) (**A**–**D**) or non-competitive ligands (fetuin, poly cytosine (pC)) (**E**–**H**) for 30 min. FV3 was added at an multiplicity of infection MOI of 1.0 for 2 h. The FV3 DNA viral loads were determined by absolute qPCR. Data are presented as means ± SEM (*n* = 3). Statistical analysis using a one-way ANOVA test and Tukey’s multiple comparisons test with 95% confidence intervals was performed. *p* values less than 0.05 are considered statistically significant. Groups with different letters are statistically different from one another.

When tadpole cells from the American toad, bullfrog, green frog, and wood frog were treated with SR-A competitive ligands (fucoidan, poly inosine (pI)), the cell-associated viral loads were drastically reduced, as compared to FV3-exposed cells that were not incubated with these ligands (Figure 1A–D), suggesting that the SR-A competitive ligands blocked FV3 viral entry. When SR-A competitive ligands were replaced with the SR-A non-competitive ligand counterparts (fetuin, poly cytosine (pC)), the cell-associated viral loads were not altered, as compared to FV3-infected cells without the ligands (Figure 1E–H). This indicates that unlike SR-A competitive ligands, SR-A non-competitive ligands did not compete for SR-A binding and did not block FV3 entry (Figure 1). In turn, these results strongly suggest that FV3 utilizes SR-As during its entry process in these tadpole cells. Because all four tadpole cell lines are MARCO-negative and WoodTad-rpe does not express SR-AI (Table 2), this suggests that SCARA3, 4, and 5 potentially have the capacity to bind FV3. However, these results do not rule out the possibility that SR-AI and MARCO may function as cellular receptors for FV3 when present. Indeed, in mammals, the ability of SR-A to bind nucleic acids was compensatory, with all members contributing to nucleic acid binding [27]. Thus, it may be that all SR-A family members contribute to FV3 entry, or there may be only a subset within the family with this ability. Further studies are needed to define the roles of specific SR-As in FV3 entry into distinct cell types. 

**Figure 2:**
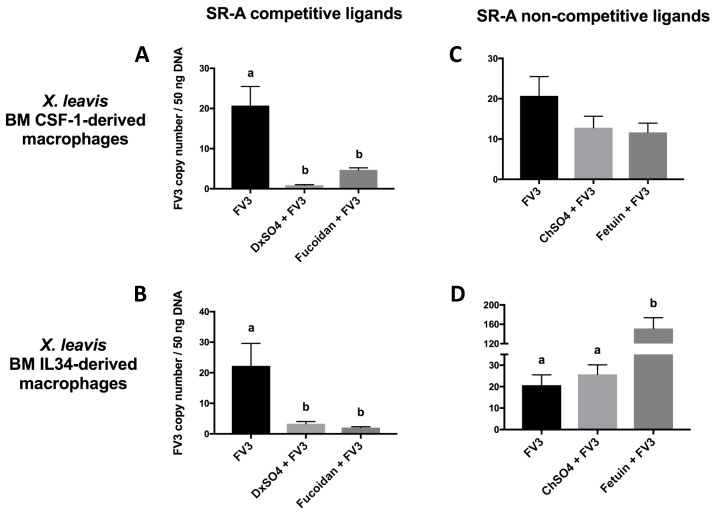
Effects of SR-A competitive and non-competitive ligands on the cell-associated FV3 load in *X. laevis* CSF-1 Mϕs and IL-34 Mϕs. The *X. laevis* CSF-1 Mϕs and IL-34 Mϕs were derived by culturing adult frog bone marrow cells with recombinant CSF-1 or IL-34, respectively, for 5 days. The Mϕs were then pre-treated with 200 μg/mL of the SR-A competitive ligands (DxSO_4_, fucoidan) (**A**,**B**) and non-competitive ligands (ChSO_4_, fetuin) (**C**,**D**) for 1 h and challenged with FV3 (MOI of 0.5) for 2 h. The FV3 DNA viral loads were determined by absolute qPCR. The results are presented as means ± SEM (*n* = 3–5). Statistical analysis using a one-way ANOVA test and Tukey’s multiple comparisons test with 95% confidence intervals was performed. *p* values less than 0.05 are considered statistically significant. Groups with different letters are statistically different from one another.

The effects of SR-A competitive and non-competitive ligands on blocking FV3 during the viral entry process were also studied in the *X. laevis* FV3-susceptible CSF-1-Mϕs and the FV3-resistant IL-34-Mϕs [7]. Notably, these Mϕ populations are equally permissive to viral entry, while the IL-34-Mϕ subset is more effective at eliminating the invading virus [5,6]. Corroborating the tadpole cell line data, two SR-A competitive ligands (dextran sulfate (DxSO_4_), fucoidan) significantly reduced the FV3 viral loads in the CSF-1-Mϕs, whereas the corresponding SR-A non-competitive ligands (chondroitin sulfate (ChSO_4_), fetuin) did not (Figure 2A,B). Similarly, DxSO_4_ and fucoidan significantly reduced the FV3 viral loads within the IL-34-Mϕs, whereas ChSO_4_ and fetuin did not (Figure 2C,D). Interestingly, the fetuin treatment led to an increase in cell-associated viral loads (Figure 2D). The reason for this unusual observation currently remains unclear and further study is needed to elucidate the underlying mechanism.

**Figure 3:**
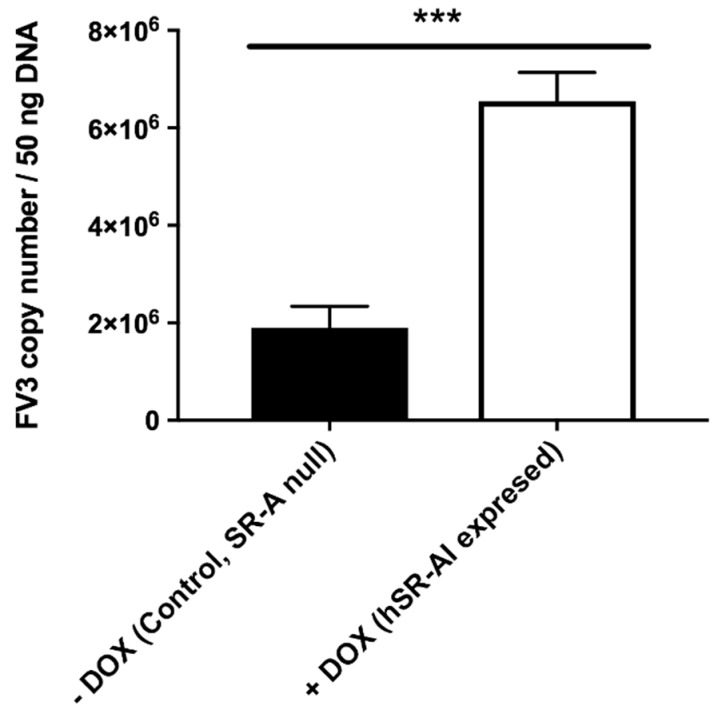
Effect of induced human SR-AI (hSR-AI) expression on the cell-associated FV3 loads in SR-A-null A549 cells. hSR-AI expression was induced with 200 ng/mL doxycycline (DOX). Control SR-A-null and DOX-treated hSR-AI-expressing cells were infected with FV3 at an MOI of 0.5 for 1 h. Total DNA was subsequently harvested and subjected to absolute qPCR analyses for FV3 DNA loads. Data are presented as means ± SEM (*n* = 3). Statistical analysis using a Student’s *t*-test with Welch’s correction was performed. ***p < 0.001.

To confirm the involvement of SR-As during the FV3 cellular entry process, we induced the expression of a human (h)SR-AI in an SR-A-null variant A549 cell line (Table 2) and examined the extent of FV3 uptake into the control and hSR-AI-overexpressing cells (Figure 3). Consistent with the data generated from the frog cells, the FV3 loads were significantly greater (approximately threefold) in cells overexpressing hSR-AI, as compared to control cells lacking SR-As. Thus, hSR-AI expression promoted FV3 uptake during the viral entry process. These findings also highlight the ubiquitous nature of SR-As, as SR-As enhanced FV3 entry in both human and frog cells (representing both non-host and host species) and at a diverse range of temperatures (37 °C and 25 °C). 

We acknowledge that our current study does not discriminate between the enveloped and non-enveloped forms of FV3 and thus, we cannot discern which form(s) of this pathogen utilize SR-As for cellular entry. As SR-As bind molecules that are negatively charged [26], the viral surface components that could interact with SR-As would very likely be coated with negatively charged functional groups. It is worth noting that SR-As appeared to mediate entry regardless of the cell source from which the virus was propagated (mammalian or piscine), indicating that the virus surface protein interacting with the SR-A may be either (1) a virus-encoded protein in the envelope; (2) a ubiquitously expressed host molecule conserved between mammals and fish; or (3) a component of the viral capsid in non-enveloped virus particles. Future studies are needed to determine which scenario is true, and should begin by investigating the recently discovered Rana grylio virus 43R envelope protein, which has been shown to be involved in Rana grylio virus entry [52].

The ubiquitous nature of SR-A expression [27] correlates with the promiscuous nature of FV3 infections. While there are likely other cellular receptors that facilitate FV3 entry, our present findings suggest that SR-As are one of the surface molecules that FV3 may utilize to gain entry into host cells. In turn, this work contributes to the broader understanding of host–ranavirus interactions [53,54], which represent valuable findings, especially in the current context of global amphibian population declines.

## Figures and Tables

**Table 1:** Cell lines and primary cultures used for frog virus 3 (FV3) infection.

Name	Cell Culture Type	Species	Developmental Stage	Cell Morphology	FV3 Isolate for Infection
BufoTad	Cell line	American toad (*Anaxyrus americanus*)	Tadpole (Gosner stage 35-38)	Endothelial-like	EPC-derived isolate
BullTad-leg	Cell line	Bullfrog (*Lithobates catesbeianus*)	Tadpole (Gosner stage 40-41)	Fibroblastic	EPC-derived isolate
GreenTad-HF2	Cell line	Green frog (*Lithobates clamitans*)	Tadpole (Gosner stage 25-27)	Fibroblastic	EPC-derived isolate
WoodTad-rpe	Cell line	Wood frog (*Lithobates sylvaticus*)	Tadpole (Gosner stage 43-45)	Epithelial	EPC-derived isolate
Bone marrow r*Xl*CSF-1-derived macrophages (Mϕs)	Primary culture	African clawed frog (*Xenopus laevis*)	Adult	Macrophage	Baby hamster kidney (BHK-21)-derived isolate
Bone marrow r*Xl*IL-34-derived Mϕs	Primary culture	African clawed frog (*Xenopus laevis*)	Adult	Macrophage	BHK-21-derived isolate
Class A scavenger receptor (SR-A)-null variant A549 cell line with an inducible human SR-AI	Cell line	Human (homo sapiens)	n/a	Epithelial	EPC-derived isolate

n/a: non-applicable.

**Table 2:** Profiles of SR-A gene expression in tadpole-derived cell lines.

Cell Cultures	SR-A Gene Expression				
	SR-AI	MARCO	SCARA3	SCARA4	SCARA5
BufoTad	positive	negative	unknown	unknown	unknown
BullTad-leg	positive	negative	positive	positive	positive
GreenTad-HF2	positive	negative	positive	positive	positive
WoodTad-rpe	negative	negative	positive	positive	positive
*X. laevis* bone marrow r*Xl*CSF-1-derived Mϕs	negative	positive	positive	positive	negative
*X. laevis* bone marrow r*Xl*IL-34-derived Mϕs	negative	positive	positive	positive	negative
SR-A-null variant A549 cell line with an inducible human SR-AI	negative but inducible	negative	negative	negative	negative
